# Single-Shot Ultra-Widefield Polarization-Diversity Optical Coherence Tomography for Assessing Retinal and Choroidal Pathologies

**DOI:** 10.3390/jcm13185415

**Published:** 2024-09-12

**Authors:** Tiffany Tse, Hoyoung Jung, Mohammad Shahidul Islam, Jun Song, Grace Soo, Khaldon Abbas, Shuibin Ni, Fernando Sumita, Katherine Paton, Yusi Miao, Yifan Jian, Zaid Mammo, Eduardo V. Navajas, Myeong Jin Ju

**Affiliations:** 1 School of Biomedical Engineering, Faculty of Medicine and Applied Science, University of British Columbia, Vancouver, BC V6T 1Z3, Canada; tse.tiffany@ubc.ca (T.T.); mohammad.islam@ubc.ca (M.S.I.); juns01@student.ubc.ca (J.S.); gracesoo@student.ubc.ca (G.S.); 2 Faculty of Medicine, University of British Columbia, Vancouver, BC V6T 1Z3, Canada; hoyoungj@student.ubc.ca (H.J.); kfabbas@student.ubc.ca (K.A.); 3 Casey Eye Institute, Oregon Health & Science University, Portland, OR 97239, USA; nis@ohsu.edu (S.N.); jian@ohsu.edu (Y.J.); 4 Department of Ophthalmology and Visual Sciences, University of British Columbia, Vancouver, BC V6T 1Z3, Canadayusi.miao@ubc.ca (Y.M.); zaid.mammo@ubc.ca (Z.M.); eduardo.navajas@ubc.ca (E.V.N.)

**Keywords:** optical coherence tomography, ophthalmology, retinal imaging, wide-field OCT, retinal diseases, polarization

## Abstract

**Background**: Optical coherence tomography (OCT) is a leading ocular imaging modality, known for delivering high-resolution volumetric morphological images. However, conventional OCT systems are limited by their narrow field-of-view (FOV) and their reliance on scattering contrast, lacking molecular specificity. **Methods**: To address these limitations, we developed a custom-built 105^∘^ ultra-widefield polarization-diversity OCT (UWF PD-OCT) system for assessing various retinal and choroidal conditions, which is particularly advantageous for visualizing peripheral retinal abnormalities. Patients with peripheral lesions or pigmentary changes were imaged using the UWF PD-OCT to evaluate the system’s diagnostic capabilities. Comparisons were made with conventional swept-source OCT and other standard clinical imaging modalities to highlight the benefits of depolarization contrast for identifying pathological changes. **Results**: The molecular-specific contrast offered by UWF PD-OCT enhanced the detection of disease-specific features, particularly in the peripheral retina, by capturing melanin distribution and pigmentary changes in a single shot. This detailed visualization allows clinicians to monitor disease progression with greater precision, offering more accurate insights into retinal and choroidal pathologies. **Conclusions**: Integrating UWF PD-OCT into clinical practice represents a major advancement in ocular imaging, enabling comprehensive views of retinal pathologies that are difficult to capture with current modalities. This technology holds great potential to transform the diagnosis and management of retinal and choroidal diseases by providing unique insights into peripheral retinal abnormalities and melanin-specific changes, critical for early detection and timely intervention.

## 1. Introduction

Optical coherence tomography (OCT) has emerged as a cornerstone in ophthalmic diagnosis and in the ongoing evaluation of treatments for various ocular conditions, given its non-invasive nature and ability to produce high-resolution depth-resolved images [1]. Since its widespread adoption, OCT technology has significantly evolved, achieving notable improvements in acquisition speed, higher-resolution cross-sectional imaging at greater depths [2], and an expansion of the field of view (FOV) [3,4,5,6]. Although OCT offers high-resolution three-dimensional images of the retina, it is limited to providing solely structural information based on light scattering properties of tissues and lacks molecular-specific contrast.

Polarization-sensitive OCT (PS-OCT) is a functional extension of OCT that enables non-invasive molecular contrast imaging in the back of the eye by detecting tissue polarization properties [7,8,9]. Melanin is a pigment molecule found in the iris, ciliary body, and retinal pigment epithelium (RPE) [10], and it is unique because it depolarizes light, which can be detected by the PS-OCT via a measure known as the degree of polarization uniformity (DOPU). By measuring DOPU, which decreases proportionally as melanin concentration increases [11,12], we can quantify and visualize the amount to which light depolarizes after interaction with the sample [8]. Polarization-diversity OCT (PD-OCT) [13,14] is a subset of the PS-OCT that offers a simpler approach for clinical deployment, capable of obtaining both DOPU contrast as well as scattering information from conventional OCT imaging. This integrated approach makes PD-OCT a promising tool for molecular contrast imaging, enabling precise assessment of abnormal melanin distribution within the retina.

According to the International Widefield Imaging Study Group, “widefield” retinal images depict retinal anatomy beyond the posterior pole but posterior to the vortex vein ampulla in all four quadrants, whereas “ultra-widefield” (UWF) images depict retinal anatomic features anterior to the vortex vein ampullae in all four quadrants [15]. Similarly, the Diabetic Retinopathy Clinical Research network (DRCR.net) has established that a threshold FOV greater than 100^∘^ is to be considered UWF [16]. As most commercially available OCT systems are generally limited to a FOV of 30^∘^ [17], acquisition of a wider view of the retina requires stitching or montaging multiple OCT scans. This approach is time-consuming, computationally expensive, prolongs image acquisition duration, and is susceptible to alignment artifacts [18].

However, despite these inherent complexities associated with UWF and WF imaging, the ability to capture the peripheral retina remains an advantageous diagnostic tool, particularly for patients with conditions involving peripheral retinal or choroidal abnormalities [19]. UWF fundus imaging and angiography have demonstrated increased detection of disease manifestations in the peripheral retina in cases of diabetic retinopathy and retinal vein occlusion [20]. Even in age-related macular degeneration, a pathology known to affect the central retina primarily, UWF imaging has demonstrated peripheral retinal findings distinct from controls. Expanding retinal imaging to include the peripheral retina may thus elucidate different phenotypic presentations of age-related macular degeneration, and the clinical significance of these peripheral findings are an area of ongoing study [21]. The volumetric information provided by UWF OCT imaging has also demonstrated peripheral retinal manifestations in disease processes affecting the peripheral retina such as retinoblastoma, retinitis pigmentosa, and multiple evanescent white dot syndrome [22,23,24]. Thus, UWF imaging of the retina may provide further insights into the pathophysiology and characterization of various retinal conditions, as well as aid in their diagnosis and clinical monitoring.

In this paper, we introduce our ultra-widefield PD-OCT system which integrates 105^∘^ FOV melanin-specific molecular contrast imaging with scattering information from conventional OCT.

## 2. Methods

### 2.1. Patient Recruitment and Data Collection

We performed an observational case-series study at three high-volume ophthalmology clinics in the Vancouver General Hospital (Vancouver, BC, Canada). Patients were given dilating eye drops (using 2.5% phenylephrine hydrochloride and 1% tropicamide) and underwent imaging with fundus photography using Topcon TRC-50DX (Topcon Corporation, Tokyo, Japan), scanning laser ophthalmoscopy (SLO) using Optos P200dTx, (Optos Inc., Marlborough, MA, USA), short-wavelength auto-fluorescence (SW-AF) at 488 nm and 532 nm excitation wavelengths using Optos P200dTx, (Optos Inc., Marlborough, MA, USA) or Heidelberg Spectralis (Heidelberg Engineering Gmbh, Heidelberg, Germany), and a research-prototype PD-OCT. All patients were informed of the purpose and implications of the study, and written informed consent was obtained from each participant prior to participation. This study was approved by the research ethics board at the University of British Columbia (human ethics protocol H19-03110 and H21-01337) and followed the tenets of the Declaration of Helsinki.

### 2.2. System Configuration and Imaging Protocol

**Figure 1:**
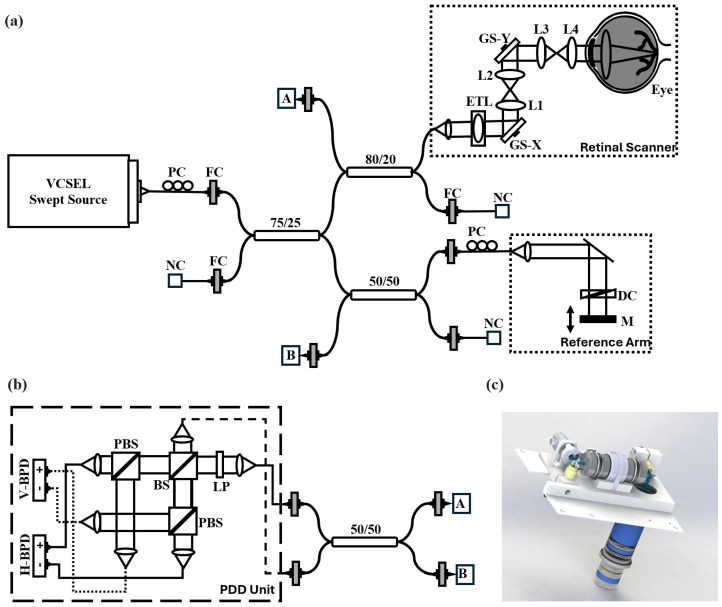
(**a**) Schematic diagram of PD-OCT system. (**b**) Polarization diversity detection unit (PDD). (**c**) SolidWorks design of the ultra-wide-field retinal scanner. L1–4: Lens; LP: linear polarizer; PC: polarization controller; FC: fiber collimator; NC: not connected; M: mirror; GS-X and -Y: galvanometer scanner; DC: dispersion compensation block; ETL: electrically tunable lens; PBS: polarizing beam splitter; BS: beam splitter; H- and V-BPD: balanced photo-detector for horizontally and vertically polarized signals, respectively.

Figure 1 represents the schematic of the PD-OCT system. A vertical-cavity surface-emitting laser (VCSEL, SVM10F-0210; Thorlabs, Inc., Newton, NJ, USA) with 100 nm bandwidth, 400 kHz A-scan rate, and a center wavelength of 1060 nm, is used as a light source. The single-mode optical fiber is used to build the interferometer. The light is split by a 75:25 single-mode optical fiber coupler after passing through the polarization controller, where 25% of the light goes to the reference arm, which consists of a fiber collimator, a dispersion compensation block, and a mirror, after passing through a 50:50 fiber coupler. The remaining 75% of the light from the coupler goes to the sample arm through the 20% of the 80:20 fiber coupler. The sample arm is attached with the custom-built retinal scanner designed and adapted from the 55^∘^ FOV handheld OCT scanning head previously published [25], which can image up to 105^∘^ FOV. The retinal scanner consists of a dual-axis galvanometer, a scan lens consisting of a paired achromatic doublet, and an ocular lens comprising an advanced double aspheric lens. Together, the scan lens and ocular lens function as a telescope, amplifying the scanning angle to about 105^∘^ at the pupil. The distance from the last lens surface to the cornea is approximately 5 mm, which has a shorter working distance than the previous clinical PD-OCT design [14] to secure a larger FOV. The beam diameter at the pupil is measured to be 0.58 mm, with sample power below 2.0 mW at the cornea, which satisfies the safety standard defined by ANSI. The backscattered light from the sample and reference are recoupled to the 80:20 and 50:50 fiber couplers, respectively, where both 80% and 50% light is directed to the polarization diversity detection (PDD) unit followed by another 50:50 fiber coupler. The PDD unit consists of a linear polarizer (LP), two polarizing beam splitters (PBS), one non-polarizing beam splitter (BS), and two 2.5 GHz balanced photodetectors (PDB 482C-AC).

In the PDD unit, the reference and back-scattered light from the eye is combined at the BS, split into horizontal and vertical polarization components by the two PBSs, and finally detected by the balanced photodetectors (BPDs). The detected signal at the BPD is digitized by a 12-bit waveform digitizer (ATS9373, AlazarTech Inc., Pointe-Claire, QC, Canada), allowing for dual-channel acquisition at a rate of 1.8 gigasamples/second in each channel. The sampled interference signal was rescaled to the wavenumber domain using a predefined rescaling parameter obtained via a time-frequency calibration method [26]. The theoretical axial resolution is 7.06 μm in air. Bi-directional high-speed raster scanning centered around the fovea is employed, which results in volume acquisitions consisting of 2304 points per A-scan, 2000 A-lines per B-scan, and 1000 B-scans per volume, yielding a total acquisition time of 5 s per volume. For the patient imaging, a total of 10 volumes were acquired per affected eye, where 5 volumes are acquired with 55^∘^ FOV, and 5 volumes are acquired with 105^∘^ FOV. The PDD unit implemented in the PD-OCT enables the computation of the degree of polarization uniformity (DOPU) [27] by measuring the orthogonally polarized beam state (horizontal-H and vertical-V channels). The contrast between these channels determines the extent to which the polarization state of the input beam is preserved after interaction with the sample, where 1 indicates full preservation (i.e., no change in polarization state), and 0 indicates no preservation (i.e., completely randomized state).

### 2.3. Field-of-View Characterization

**Figure 2:**
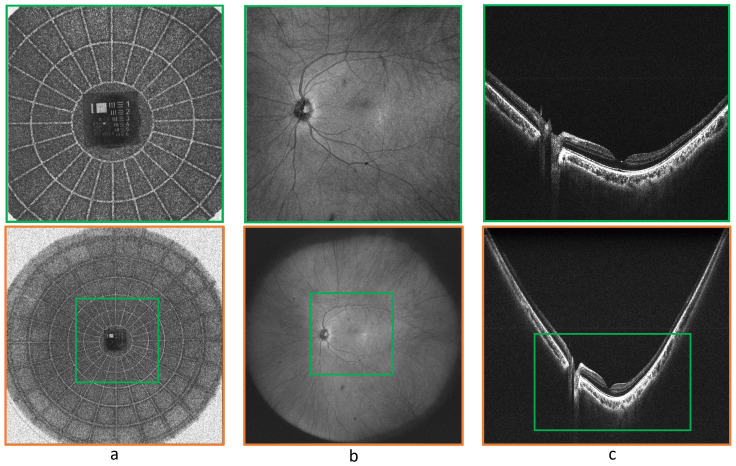
Comparative images of two distinct FOVs (55^∘^ and 105^∘^). (**a**) Phantom eye featuring a resolution target with structured concentric circles that serve as a visual guide for FOV and resolution calibration. (**b**,**c**) OCT en face and B-scan images of healthy control subject where the green rectangular box highlights the 55^∘^ FOV and the encompassing orange box designates the 105^∘^ FOV.

The uniformity, continuity, and absence of irregularities in tissue layers, even at the peripheral sections, serve as qualitative indicators of good ocular health. Figure 2 presents a comparative analysis of the imaging capabilities of our custom-built retinal scanner, emphasizing the differences in FOV of 55^∘^ versus 105^∘^. Figure 2a depicts the phantom eye (Rowe Technical Design, Inc., Dana Point, CA, USA) embedded with a resolution target, where the structured concentric circles on the target facilitate the calibration of resolution and provide a visual guide for evaluating the performance of scanner across different FOVs. The green and orange boxes represent the areas covered by the 55^∘^ and 105^∘^ FOVs, respectively, highlighting the extended imaging capability at the larger FOV. Figure 2b,c show the *en-face* and B-scan images of a healthy control patient’s retina, where the green rectangular box marks the area encompassed by the 55^∘^ FOV, and the orange box indicates the significantly wider area covered by the 105^∘^ FOV. The images clarify how the panoramic FOV can capture a greater extent of the peripheral retinal structure, which is crucial for a thorough retinal examination and for the detection of peripheral retinal pathologies.

### 2.4. Post-Processing and Feature Extraction

**Figure 3:**
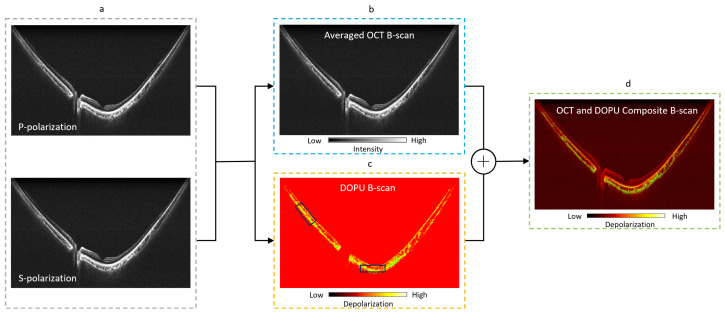
Illustration of post-processing pipeline: (**a**) raw OCT intensity images detected by the PDD unit of P-polarization (horizontal) and S-polarization (vertical) channels; (**b**) scattering OCT B-scan obtained by coherent averaging of P- and S-channels; (**c**) noise-corrected DOPU B-scan kernel averaging [27]; (**d**) OCT and DOPU B-scan composite.

Following data acquisition, the post-processing pipeline used for generating multi-contrast images is illustrated by Figure 3. The complex OCT volume (Figure 3a) is derived from the two orthogonally polarized channels using conventional OCT pre-processing steps: Hilbert transform, wavenumber linearization [26], DC subtraction, numerical dispersion compensation [28], and fast Fourier transform. The contrast-enhanced scattering OCT volume is obtained by averaging the complex data (Figure 3b). To account for additive noise inherent in each polarization channel, the noise-induced error is estimated from the PD-OCT signals to obtain noise-error-corrected Stokes parameters [27]. The noise-corrected DOPU is calculated as a measure of the variance in Stokes vectors averaged within a localized area, highlighting variations in polarization state between neighboring pixels (Figure 3c) [11,27,29]. To optimize the contrast and sharpness of DOPU images, a 3×5 pixel averaging kernel is employed. Subsequent thresholding is applied to the DOPU values, which are then smoothed by a three-dimensional median filter (3×5×3 pixels) for removal of any remaining background noise. The composite B-scan is created from averaged OCT and DOPU B-scans (Figure 3d) to provide a distribution map of tissue along the retina with polarization-scrambling properties.

For visualization, the RPE layer is manually segmented from each volume based on OCT B-scan images via a segmentation assistant software (ITK-Snap [30]) to distinguish melanin content between the RPE, choroid, and inner retina. The minimum projection of the final DOPU B-scans are taken along the depth axis to generate DOPU en face maps, providing a comprehensive view of the polarization properties across the retinal layers.

## 3. Results and Discussion

### 3.1. Retinitis Pigmentosa

Retinitis pigmentosa (RP) is the most common hereditary retinal dystrophy, with a prevalence of approximately 1 in 4000 individuals [31]. It refers to a heterogenous group of retinal diseases characterized by the degeneration of the RPE, rod photoreceptors, and cone photoreceptors [32,33]. The subsequent visual field losses correlate with the loss of photoreceptor cells, which typically affects the mid-peripheral retina and progresses towards the far periphery and macula [31]. The utilization of fundus photographs, fundus autofluorescence (FAF), and OCT imaging modalities has been the cornerstone of the diagnosis and monitoring of retina disease progression [34]. In RP, OCT assesses retinal thickness, ellipsoid zone (EZ) line width, and the presence of macular edema [31,35]. EZ disruptions correlate with lower visual acuity and other functional impairments [36]. Hyperautofluorescence on SW-AF represents lipofuscin in the RPE, a byproduct of photoreceptor metabolism, marking the transition between healthy and degenerating retinas in RP [12,32,35,37].

**Figure 4:**
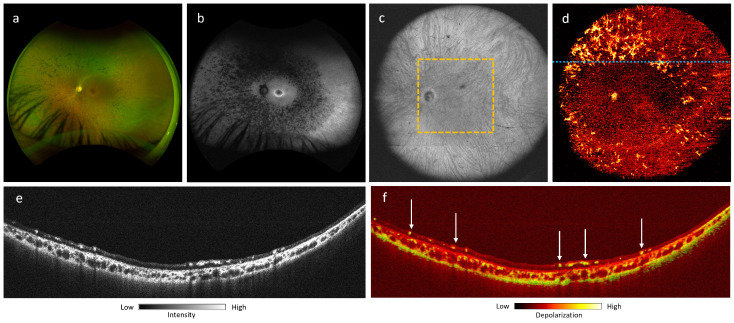
Retinitis pigmentosa patient, a 38-year-old South Asian male with heterozygous RHO GLu181Lys genetic mutation (left eye): (**a**) SLO fundus photograph; (**b**) short-wavelength autofluorescence; (**c**) 105^∘^ ultra-wide FOV OCT *en-face* projection image captured by PD-OCT; (**d**) 105^∘^ ultr-awide FOV DOPU *en-face* projection image of the inner retina after segmentation; (**e**,**f**) 105^∘^ FOV OCT and DOPU B-scans at location marked by dotted blue line in (**d**). Bone spicules in the peripheral retina are highlighted by the white arrows in (**f**).

**Figure 5:**
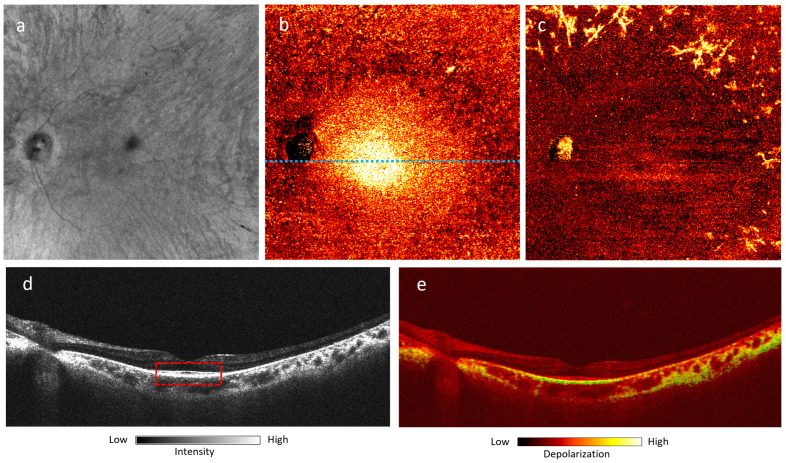
Close-up 55^∘^ FOV marked by the yellow dashed square in Figure 4c: (**a**) OCT *en-face* projection image captured by PD-OCT; (**b**) DOPU *en-face* projection image of the RPE after segmentation; (**c**) DOPU en face projection image of the inner retina after segmentation; (**d**,**e**) OCT and DOPU B-scans at location marked by dotted blue line in (**b**). The ellipsoid zone is highlighted by the dotted red rectangle in (**d**).

Figure 4 shows a case of RP associated with a heterozygous RHO GLu181Lys genetic mutation imaged with UWF PD-OCT. We compared these results with conventional imaging techniques like SLO fundus and SW-AF (Figure 4a,b). Figure 5 is a close-up view of the area marked by the yellow dashed square in Figure 4c. En face projection of the RPE layer (Figure 5b) demonstrated higher depolarization, representing melanin content near the macula, consistent with the region of hyperautofluorescence on FAF (Figure 4b). There was a notable absence of melanin signal in the peripheral RPE, corresponding to the regions with bone spicule pigmentation on fundus photography (Figure 4a). OCT and DOPU B-scans (Figure 5d,e) highlight the absence of melanin signal in the peripheral RPE, whereas melanin signal is still preserved at the macula. The bone spicule pigmentation in Figure 4d appears in the same pattern as in Figure 4a, consistent with the migration of pigment cells from RPE to the inner retina. This pattern is visible in peripheral B-scan locations, marked by areas of high depolarization (Figure 4e,f).

Our findings have demonstrated that ultra-wide FOV allows for the robust evaluation of the central and peripheral retina and choroid while avoiding the need for multiple acquisitions and post-acquisition montaging. This is notable considering that patients with significant peripheral visual field deficits in late-stage disease can have difficulty in identifying fixation targets during multiple serial acquisitions. In the case of RP, the degenerative processes affecting the photoreceptors and RPE typically initiates in the peripheral retina before migrating centrally; thus, ultra-wide FOV imaging can be useful in detecting the early stages of degeneration affecting the peripheral retina. This can be especially beneficial for screening asymptomatic patients with family history of RP. As features such as melanin loss in the RPE and the presence of melanin in the inner retina can be characterized by PD-OCT, melanin may be a potential biomarker in monitoring treatment responses and the efficacy of gene therapy.

### 3.2. Choroidal Nevi

Choroidal nevi represent the most common benign intraocular tumor, with a prevalence of approximately 5%, although this varies by ethnicity [38,39]. Nevi are estimated to have an annual 1 in 8845 risk for malignant transformation into melanoma [40]. Routine monitoring via multimodal imaging via fundus photography, FAF, OCT, and ultrasonography are thus essential in the evaluation of these tumors [41].

**Figure 6:**
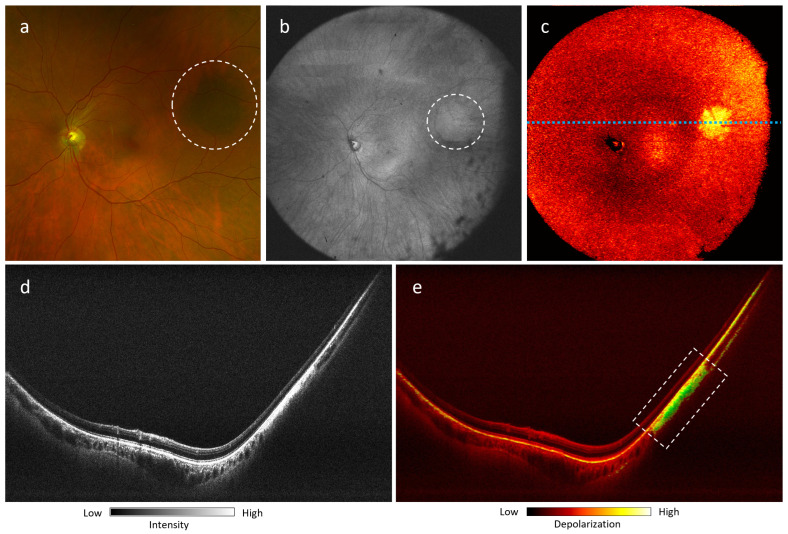
Peripheral flat melanotic choroidal nevus of a 55-year-old Caucasian female (left eye): (**a**) fundus photograph with lesion highlighted by white dotted circle; (**b**) 105^∘^ ultra-wide FOV OCT *en-face* projection with lesion highlighted by white dotted circle; (**c**) 105^∘^ ultra-wide FOV DOPU *en-face* projection; (**d**,**e**) OCT B-scan and DOPU B-scan of lesion at location marked by blue dotted line in (**c**). The area of depolarization consistent with the melanin-rich area is highlighted by a white dotted rectangle in (**e**).

In Figure 6, we present a flat melanotic choroidal nevus with a pigmented appearance on the fundus photograph (Figure 6a). The margins of lesion are not readily apparent on OCT en face projection (Figure 6b), whereas DOPU en face and B-scan projections of the lesion demonstrate a well-circumscribed melanotic appearance (Figure 6c,e), consistent with its fundus pigmentation.

**Figure 7:**
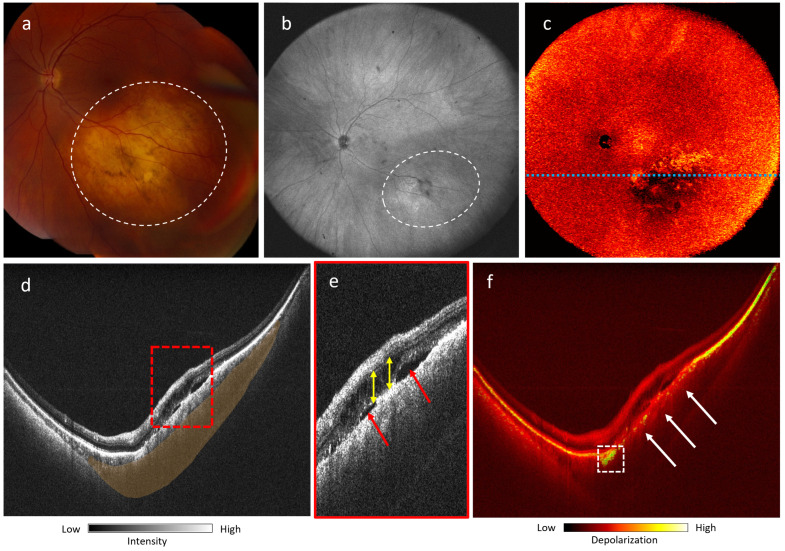
Mixed melanotic/amelanotic choroidal nevus of a 53-year-old Caucasian female (left eye): (**a**) Fundus photograph with lesion highlighted by white dotted circle; (**b**) 105^∘^ ultra-wide FOV OCT *en-face* projection with lesion highlighted by white dotted circle; (**c**) 105^∘^ ultra-wide FOV DOPU *en-face* projection. (**d**) OCT B-scan of lesion at location marked by dotted blue line in (**c**); Retinal elevation highlighted in orange; (**e**) Magnified view of area highlighted by red dotted rectangle in (**d**), showing subretinal fluid (red arrows) and intraretinal fluid (yellow arrows); (**f**) DOPU B-scan of lesion at location marked by dotted blue line in (**c**). A strong melanin signal at the edge of the lesion is indicated by a dotted white rectangle, and loss of melanin signal at the RPE is indicated by white arrows.

Figure 7 shows a larger choroidal nevus, for which full margin acquisition with fundus photograph would require montaging (Figure 7a), whereas PD-OCT can acquire full lesion margins in a single acquisition (Figure 7b,c). Retinal elevation due to the mass effect of the lesion can be visualized fully via the B-scan, with clear visualization of subretinal fluid and intraretinal fluid (Figure 7d,e). The lesion appears mostly non-pigmented on the fundus photograph, although the lesion has a mixed melanotic/amelanotic appearance on the DOPU B-scan, showing a focal region of melanin (Figure 7f) with a marked absence of melanin in the RPE and a primarily amelanotic mass body.

We have demonstrated that depth-resolved melanin-specific contrast can provide an additional objective characteristic in the clinical evaluation of choroidal tumors. For melanotic lesions, PD-OCT enables the delineation of axial and longitudinal tumor margins based on melanin signal. PD-OCT also enables the objective assessment of lesion pigmentation. Furthermore, with an expanded FOV of 105^∘^, PD-OCT enables a more comprehensive view of large lesions and can capture its entirety in a single acquisition, thereby enhancing the precision of basal diameter and thickness measurements. This can assist with the serial growth monitoring of nevi and the evaluation of morphologic and melanin-specific changes over time or in response to anti-melanoma treatment.

### 3.3. Multifocal Choroiditis

Multifocal choroiditis (MFC) is a non-infectious, idiopathic disorder that was described by Dreyer and Gass in 1984 [42]. The disease presents with “punched-out” atrophic chorioretinal scars of variable size, typically greater than 250 μm, as well as an anterior chamber and vitreous inflammation. Although MFC is similar to presumed ocular histoplasmosis syndrome, its description includes no evidence of prior histoplasmosis infection. Its incidence is estimated at 0.03 cases per 100,000 people per year [43], and the disease predominantly affects Caucasian women with myopia in their second-to-fourth decade of life [44]. Multimodal analysis findings are well described in the medical literature and include fundus photographs, fluorescein angiography, FAF, and OCT [45]. The lesions are typically at the level of the outer retina, RPE, and choriocapillaris. During the inactive stage of the disease, the lesions demonstrates RPE disruption, and the absence of an ellipsoid zone is common [45]. On FAF, the lesions show a hypoautofluorescence pattern. Active lesions have an hyperautofluorescent halo, which is absent during inactive disease. Symptoms include scotomas, metamorphopsia, floaters, and photopsias. Visual acuity is usually good at presentation. Curvilinear chorioretinal streaks, named Schlaegel lines, might be found in the periphery [46].

**Figure 8:**
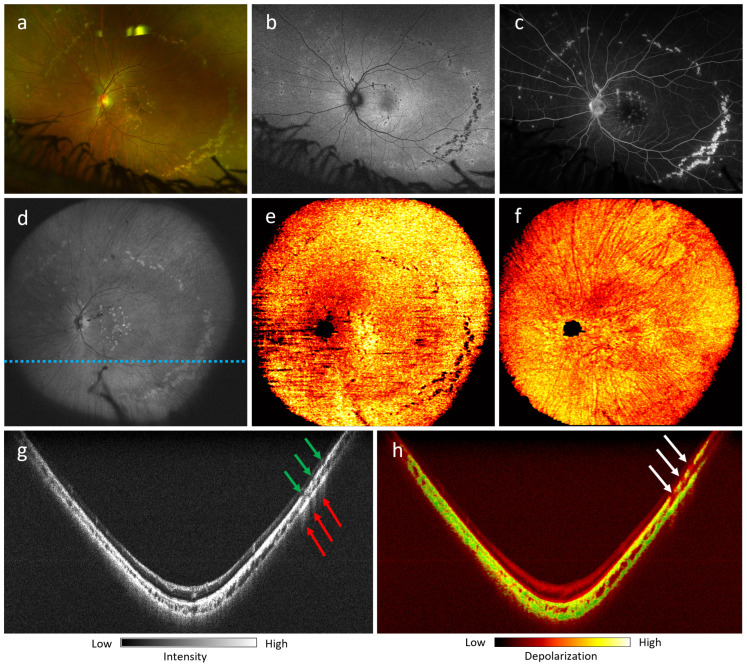
Multifocal choroiditis of a 26-year-old female. Best corrected visual acuity: 20/60. (**a**) Fundus photograph; (**b**) short-wavelength autofluorescence; (**c**) fluorescein angiography; (**d**) 105^∘^ ultra-wide FOV OCT *en-face* projection captured by PD-OCT; (**e**) 105^∘^ ultra-wide FOV DOPU *en-face* projection of the RPE; (**f**) 105^∘^ ultra-wide FOV DOPU *en-face* projection of the choroid; (**g**,**h**) 105^∘^ ultra-wide FOV OCT and DOPU B-scans at the location marked by the dotted blue line in (**d**). Green arrows mark decreased RPE reflectivity. Red arrows show increased light transmission through the choroid and sclera at the sites of the scars. White arrows show the absence of depolarization signal at the level of the RPE and choriocapillaris.

Figure 8 shows the findings for a 26-year-old female patient who had myopia and MFC. The patient complained of metamorphopsia on the left eye, with a best corrected visual acuity of 20/60. The fundus photograph (Figure 8a) demonstrates multiple punched-out atrophic chorioretinal scars in the macula and mid-periphery. In the inferotemporal quadrant, the scars group in a curvilinear shape, forming a Schlagel line. FAF demonstrates hypoautofluorescence in the center of the lesions with a hyperautofluorescent halo (Figure 8b), and fluorescein angiography shows hyperfluorescent scars (Figure 8c). Ultra-wide FOV *en-face* OCT projection (Figure 8d) presents an increased reflectivity of the lesions. The 105^∘^ ultra-wide FOV DOPU *en-face* images of the RPE (Figure 8e) and of the choroid (Figure 8f) show lesions with absent depolarization in the RPE and increased projection of DOPU in the choroid. The OCT B-scan (Figure 8g) at the level of the lesions shows decreased reflectivity of the RPE at the scars (green arrows), with hypertransmission of the light through the choroid and sclera (red arrows), while the DOPU B-scan (Figure 8h) demonstrates absent depolarization at the level of RPE and choriocapillaris (white arrows).

These findings demonstrate that UWF PD-OCT DOPU analysis is able to demonstrate depolarization changes in both RPE and choroid in patients with MFC. Moreover, this technology quantifies the degree of increased or decreased polarization in the MFC lesions and surroundings and differentiate the changes that take place in the RPE/choriocapillaris from those that occur in the choroid. As shown in the present case, MFC scars affect the RPE/choriocapillaris complex and results in decreased melanin in that layer, while the choroid remains unchanged. Such differentiation is only possible using UWF PD-OCT.

### 3.4. Choroideremia

Choroideremia is an X-linked chorioretinal dystrophy caused by a mutation in the CHM gene encoding the Rab escort protein-1 (REP-1). It is characterized by progressive degeneration of the RPE, photoreceptors, and choroid [47]. Clinical manifestations can include impaired night vision in childhood accompanied by progressive peripheral visual field loss. Choroideremia carriers can demonstrate pigmentary mottling on fundus photograph and speckled appearance of hypoautofluorescence and hyperautofluorescence on FAF, even in the absence of obvious fundus changes [48,49].

**Figure 9:**
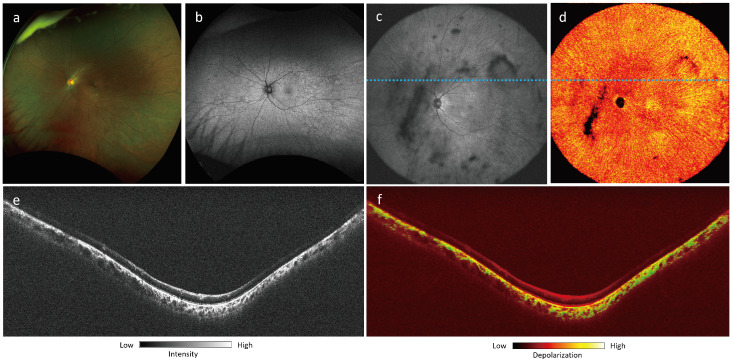
Choroideremia carrier, a 41-year-old Asian female: (**a**) fundus photograph; (**b**) short-wavelength autofluorescence; (**c**) 105^∘^ ultra-wide FOV OCT *en-face* projection; (**d**) 105^∘^ ultra-wide FOV DOPU *en-face* projection; (**e**) OCT B-scan at location marked by dotted blue line in (**c**); (**f**) PD-OCT B scan projection at location marked by dotted blue line in (**c**).

Figure 9 shows a 41-year-old female choroideremia carrier, visually asymptomatic, with 20/20 visual acuity in both eyes and normal visual fields. The fundus photograph demonstrates subtle pigment mottling throughout the mid and far periphery (Figure 9a). FAF shows a speckled pattern of hypoautofluorescence, primarily affecting the mid-peripheral retina, sparing the perifovea (Figure 9b). OCT B-scan projection (Figure 9e) demonstrates hyper-reflectivity in the choroid, following a similar pattern to the depolarization signal on the DOPU B-scan (Figure 9f), which shows increased depolarization in the mid and temporal regions. However, increased depolarization in the nasal portion is confined to the inner choroid. This finding may suggest elevated levels of choroidal melanin, although it may also be generally common for Asian patients to exhibit higher melanin content in the choroid.

Previous studies have demonstrated characteristic patterns of fluorescence and RPE mosaic in choroideremia carriers [50]. Melanin-specific contrast imaging using PD-OCT offers an additional contrast modality that can aid in the further investigation of these patterns in both choroideremia and carrier cases, whereas the speckled appearance on FAF is localized and non-specific. This provides additional insight into choroidal involvement that is not evident on fundus photography and FAF. The 105^∘^ ultra-wide FOV also allows for the observation of whole-choroidal involvement posterior to the equator. This raises the possibility of a specific choroidal melanin phenotype in carriers that could affect the entire choroid. Further PD-OCT imaging of a larger sample of choroideremia carriers and affected individuals could offer additional insights into the potential association between RPE/choroidal melanin and visual phenotype.

## 4. Discussion

In this study, we introduced a novel UWF PD-OCT system that provides depth-resolved imaging with depolarization contrast for assessing retinal and choroidal pathologies. This system was evaluated in patients with conditions such as choroidal lesions, multifocal choroiditis, and inherited retinal diseases. Our findings demonstrate the potential of UWF PD-OCT to provide comprehensive imaging of peripheral retinal abnormalities, contributing valuable insights into melanin distribution and pigmentary changes.

We demonstrated several advantages of the PD-OCT over existing imaging modalities. Compared to commercially available wide-field imaging modalities like SLO and FAF, which capture only 2D *en-face* images, PD-OCT provides depth-resolved images that can visualize retinal layers while also offering additional depolarization contrast. This capability enables the detection of melanin distribution abnormalities in ways that other OCT systems currently used in clinics cannot, as conventional OCT primarily relies on scattering contrast. Moreover, PD-OCT acquires a much larger FOV in a single shot, eliminating the need for montaging multiple scans, which not only minimizes patient discomfort and motion artifacts but also reduces computational costs. Furthermore, this technology can be used in conjunction with existing modalities in the clinic to provide complementary information. While conventional tools like fundus photography or standard OCT systems offer valuable structural insights, PD-OCT adds a unique layer of information, which can enhance diagnostic accuracy and offer new perspectives in assessing retinal pathologies. Future development of multi-modal image registration techniques, such as registering fundus images with PD-OCT *en-face* images, would offer a more comprehensive view of retinal health while maintaining consistency with imaging workflows that clinicians are already familiar with. Such multi-modal approaches would also ease the clinical translation of PD-OCT by aligning its functionality with widely accepted practices in ophthalmology.

Despite its advantages, we acknowledge several limitations of our study with the current prototype. First, while the system effectively visualizes melanin distribution through DOPU, it cannot yet quantify melanin concentration, which poses challenges in comparing melanin levels between patients. To address this, future work will focus on implementing a uniform modulation of the input polarization state, allowing us to obtain a quantifiable depolarization index with which to evaluate melanin concentration [51,52]. This enhancement will require multiple volume acquisitions and the development of a sophisticated 3D registration algorithm to align and average these volumes accurately. Second, patient movements such as microsaccades or blinking can introduce significant motion artifacts in the captured volumes, particularly in patients with low visual acuity which affects their ability to fixate, or in those with dry eyes, making it difficult for them to keep their eyes open during the acquisition process. Although 8–10 volumes are acquired per eye during each imaging session, we are developing a feature-based registration algorithm to account for geometric warping of the vasculature and mitigate information loss caused by movement. This will allow us to generate a fully motion-corrected volume, enhancing both the accuracy and reliability of the images. Lastly, it is worth noting that the selection of specific diseases for this study was based on their peripheral abnormalities and the presence of pigmentary changes, which make them suitable for demonstrating the capabilities of the PD-OCT system. However, this focus limits the generalizability of our findings to other retinal conditions that also involve peripheral lesions. In order to explore the application of PD-OCT to a broader range of retinal diseases, future work will expand its functionality to include OCT angiography (OCTA). The integration of OCTA will allow for the visualization of ultra-wide FOV vascular contrast, enabling the exploration of additional conditions such as diabetic retinopathy and glaucoma. Despite these limitations, our prototype has consistently demonstrated its ability to provide detailed morphological and melanin-contrast images with UWF retinal coverage, underscoring its potential to offer novel insights into retinal conditions that are not possible with existing clinical tools.

## 5. Conclusions

In summary, UWF imaging with PD-OCT enables robust, non-invasive visualization of melanin-specific features in the peripheral retina. Beyond providing melanin-specific contrast, UWF PD-OCT offers detailed visualization of retinal structures comparable to conventional swept-source OCT, while eliminating the need for montaging multiple acquisitions. Our findings establish melanin distribution as a critical marker for assessing retinal health, and future studies will solidify its role as a biomarker in diagnosing and evaluating retinal conditions.

## Data Availability

The datasets generated and analyzed during the current study are available from the corresponding author upon request.
